# Clean-up of divalent cobalt ions by massive sequestration in a low-cost calcium silicate hydrate material

**DOI:** 10.1038/s41598-024-56617-x

**Published:** 2024-03-25

**Authors:** Andrea Hamilton, Pieter Bots, Han Zhou, Bao Liu, Christopher Hall

**Affiliations:** 1 Department of Civil and Environmental Engineering, University of Strathclyde, Glasgow, G1 1XJ UK; 2 School of Materials Science and Engineering, Nanjing Tech University, Nanjing, 211800 China; 3 School of Materials Science and Engineering, Southeast University, Nanjing, 211189 China; 4 School of Engineering, University of Edinburgh, The King’s Buildings, Edinburgh, EH9 3JL UK

**Keywords:** Geochemistry, Porous materials, Chemical engineering, Geochemistry, Porous materials, Chemical engineering

## Abstract

Cobalt is a critical resource in industrial economies for the manufacture of electric-vehicle batteries, alloys, magnets, and catalysts, but has acute supply-chain risks and poses a threat to the environment. Large-scale sequestration of cobalt in low-cost materials under mild conditions opens a path to cobalt recycling, recovery and environmental clean-up. We describe such sequestration of cobalt by a widely available commercial calcium silicate material containing the mineral xonotlite. Xonotlite rapidly and spontaneously takes up 40 percent of its weight of cobalt under ambient conditions of temperature and pressure and reduces dissolved cobalt concentrations to low parts per million. A new Sharp Front experimental design is used to obtain kinetic and chemical information. Sequestration occurs by a coupled dissolution-precipitation replacement mechanism. The cobalt silicate reaction product is largely amorphous but has phyllosilicate features.

## Introduction

Cobalt is a vital resource that sits near the top of US, EU and UK lists of critical minerals^1–3^ ranked by supply-chain risk, and is a main target of government policy responses^4–6^. Cobalt has key uses in batteries, permanent magnets, superalloys and catalysts^7^. About 60 percent of global primary cobalt is produced in Congo-Kinshasa^8^, much of which is then refined in China^9^. Small-scale artisanal mining cruelly exploits workers and generates large amounts of toxic waste, the cause of pervasive health and ecosystem damage^10–12^.

New methods are needed urgently to remove cobalt from the environment and for end-of-life recycling. The sequestration of cobalt ions by calcium silicate hydrates is a significant discovery of practical value both in toxic waste management and in cobalt recovery. These hydrates are the main matrix minerals in widely available low-cost manufactured cement materials. Here we demonstrate the massive uptake of Co(II) ions from solution by one such manufactured material known as calcium silicate [CS] building insulation board. CS consists largely of the mineral xonotlite Ca$_{6}$Si$_{6}$O$_{17}$(OH)$_{2}$^13^. Its sequestration of Co(II) from aqueous solution is at least five times greater than that of other materials, such as clays, zeolites and carbons^14^ and several hundred times greater than its accumulation in plants and biofilms^15,16^. CS is manufactured from calcium hydroxide (hydrated lime) and silica under hydrothermal conditions^17,18^.

The immobilisation of heavy metals in calcium silicates has been investigated previously^19^, but usually as a surface sorption or ion-exchange process. Most often the immobilised metal is incorporated as a reactant in a synthesis run^20,21^, a process that does not simulate removing contaminants from water. In the 1980s there was interest in Al-substituted tobermorites as cation-exchange materials^22–24^. It was reported that Co (and Ni) in dilute solution exchanged completely with the calcium of tobermorite and xonotlite^25,26^, although in^27^ a much lower amount of exchange was found. Synthetic Al-tobermorite was considered “...useful as a scavenger for trace heavy metals”^28^. None of this work considered large-scale environmental sequestration. There was early interest in Co clays^29^, and more recently in the synthesis of Co phyllosilicates^30^ in the search for efficient catalysts for water-splitting^31^ to generate hydrogen.

The Co sequestration reaction we describe here involves complete reconstruction of the silicate matrix and is not satisfactorily described either as a sorption or as an ion-exchange process. It more closely resembles a replacement reaction in the sense recently used in geochemical mineralogy^32^.

## Results and discussion

### CS characterisation

**Figure 1:**
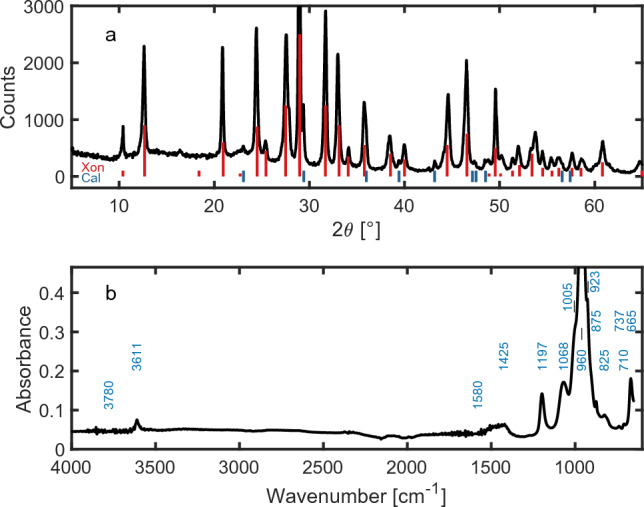
(**a**)   Powder XRD (Cu anode) pattern of CS; reference patterns of xonotlite (Xon) from ICDD 23-125, and of calcite (Cal) from ICSD 191852. (**b**)   Mid-IR spectrum of CS. The complex overlapping bands in the region 700–1200 cm^−1^ are largely due to vibrational modes of the silicate structure of xonotlite. Weak bands at 1425, 875 and 710 cm^−1^ match reference spectra of calcite^33^.

CS consists largely of the calcium silicate hydrate mineral xonotlite Ca$_{6}$Si$_{6}$O$_{17}$(OH)$_{2}$ (Xon 91.3 wt percent), with calcite (Cal 6.3 wt percent) and cellulose fibres (2.4 wt percent) as minor components (see Supplementary Information). Only xonotlite is chemically active in contact with Co solutions. Figure 1a shows the powder X-ray diffraction [XRD] pattern of CS, which apart from the absence of the weak reflection at 18.4$^\circ$ $2\theta$ is in excellent agreement with reference pattern ICDD 23-125 of an autoclaved xonotlite synthesised from lime and silica at 200 $^\circ$C  for 24 h. Xonotlite has a strongly fibrous habit^34^ with the fibre axis parallel to silicate double chains which lie along the [010] crystallographic direction^35^. Double chains are formed by bridging-oxygen crosslinks shared between every third silicate tetrahedron of individual chains. Parallel double chains then lie between sheets of Ca polyhedra in the (001) crystallographic plane. In the ideal formula, xonotlite contains no molecular water; OH groups are located in the Ca octahedra of the sheets.

The mid-IR spectrum of CS is shown in Fig. 1b. The main bands include the sharp band at 1197 cm^−1^  assigned^36,37^ to the Si-O-Si stretching mode of the crosslinks of the double chain, the intense band at 960 cm^−1^  and the weaker one at 1068 cm^−1^  assigned to Si-O-Si stretching vibrations of non-bridging tetrahedra^37^. The only feature in the water region 3000–4000 cm^−1^  is the small band at 3611 cm^−1^  assigned to the OH groups of xonotlite^38^. There is no evidence of molecular water in CS.

### Co sequestration

#### Solutions analysis

The sequestration of Co by CS was tested by reacting powdered CS with aqueous Co(NO$_3$)$_2$ solutions of initial concentration $b({\textrm{Co}})_0$ 0.35, 0.073, 0.037 and 0.0035 *m* (where *m* denotes molal concentration: mol/kg water) for periods of 2, 4, 6, 8 h, 1:14, 28 and 56 days. In all cases, 1.00 g of CS was mixed with 25.0 mL aliquots of Co solution or a control aliquot of deionised water. The highest solution concentration contained slightly more Co than needed to completely replace Ca in the available xonotlite. At each sampling time, the supernatant was analysed for the Ca and Si released from CS, and for the Co remaining in solution.

**Figure 2:**
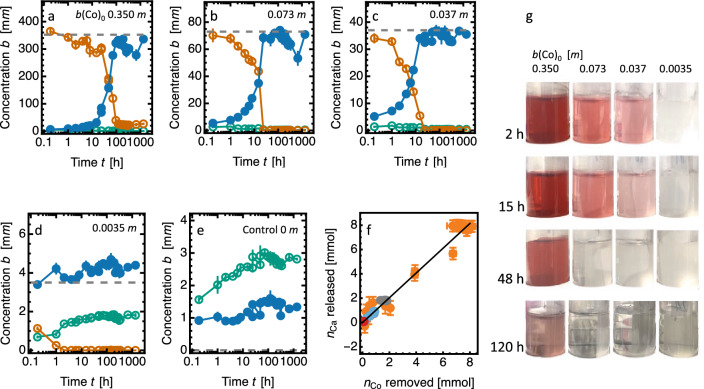
Solution composition during the Co-Ca sequestration reaction. (**a**–**e**)   Concentrations (*b*) of Ca (blue), Co (orange), Si (green); which are the means of three measurements with error bars showing the range; horizontal dotted lines show the initial Co concentration $b({\textrm{Co}})_0$. (**f**)   Amount of Ca released $n_{\textrm{Ca}}$ vs amount of Co removed $n_{\textrm{Co}}$: composite plot of data from all solutions; regression line $n_{\textrm{Ca}}= (1.02\pm 0.03) n_{\textrm{Co}}$. (**g**)  Photographs of samples (decreasing initial concentration from left to right) taken before the solutions were decanted for analysis, loss of colour highlighting the progressive removal of Co from solution.

Figure 2a–c show that the removal of Co *from* solution is accompanied by a synchronous and equivalent release of Ca *into* solution. The time for complete removal decreases with decreasing Co concentration in solution and takes roughly 100 h at the highest concentration used (0.35 *m*). At all times the concentration of dissolved Si is extremely small, generally less than $1.0 \pm 0.1$ m*m*  (30 ppm). In Fig. 2d the initial amount of Co in the solution (0.0035 *m*) is removed in less than 1 h. The final concentration of Ca in solution is close to the initial concentration of Co for all concentrations used, showing the same Co removal behaviour at both low and high concentrations. After reaction, the residual Co is extremely low. In solutions that remain in contact with excess CS, the mean Co concentration from daily measurements over the period from 4 to 9 days after first contact with CS is extremely small ($0.06 \pm 0.04$ ppm) and below the method quantification level.

The control solution (zero Co), Fig. 2e, provides a useful baseline. The concentration of dissolved Si is in the range 1.5–3.0  m*m*, while the Ca concentration is 1.0–1.5  m*m*. These low concentrations (40–80 ppm) are approximately as calculated from the known solubilities of xonotlite and calcite.

Figure 2f   shows that the amount of Co sequestered is equal to the amount of Ca released at all time steps and in all solutions. The replacement is stoichiometric throughout the reaction, with $n_{\mathrm {Ca}}/n_{\mathrm {Co}} = 1.02\pm 0.03$.

These observations show that sequestration occurs through the formation of a Co silicate phase, since the Si concentration in solution does not change during the reaction. We denote this product phase as Co-S-H, following cement chemistry notation in which S is used for SiO$_2$ and H for H$_2$O, with dashes indicating that the stoichiometry may be variable or not known.

The clean-up of the strongly coloured Co$^{2+}$ ion from solutions of different initial concentration is shown visually in Fig. 2g.

#### Characterisation of the reaction product

**Figure 3:**
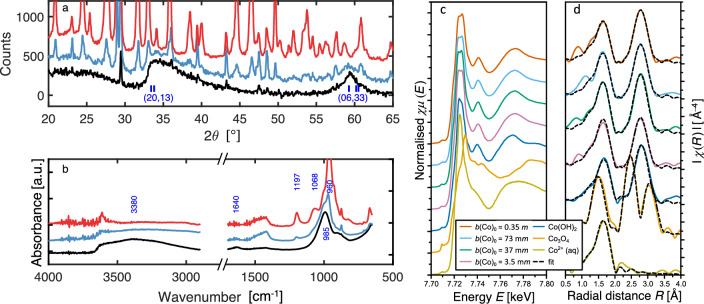
Changes in solids composition during sequestration. (**a**) XRD patterns of solid material formed by reaction of CS with Co(NO$_3$)$_2$ solution of initial concentration $b({\textrm{Co}})_0$ = 0.35 *m* after 2 h (red), 2 days (blue) and 5 days (black); tick marks show positions of Co-phyllosilicate (*hk*) reflections^39^ (also ICDD 21-871, 21-872). (**b**)   Mid-IR spectrum of the same solid material shown in (**a**). (**c**, **d**)   X-ray absorption spectra of the final product after 56 days reaction and the standards used (Co(OH)$_2$, Co$^{2+}$(aq), Co$_3$O$_4$), where $\chi \mu (E)$ is the normalised XANES (**c**), $|\chi (R)|$ is the Fourier transform of the $k^3$-weighted EXAFS $\chi (k)$ (**d**), and *k* is the wavenumber. Solid black lines (—) represent the final fits to the Fourier transform of the EXAFS (see Supplementary Information).

The product of the CS reaction with solution $b({\textrm{Co}})_0$ = 0.35 *m*, in which all Ca initially present is replaced by Co, is amorphous as shown by XRD and FTIR analyses (Fig. 3a and b).

**Figure 4:**
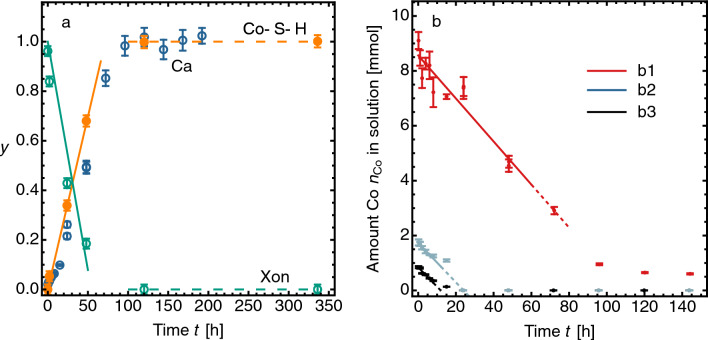
Kinetic phenomena in Co sequestration by CS: observations of stirred powder reactions (25 mL solution mixed with 1.00 g CS powder) at 25 $^\circ$C. (**a**)   Initial Co concentration ($b({\textrm{Co}})_0$ = 0.35 *m*), showing release of Ca$^{2+}$ into solution (Ca, blue points, ICP measurement), decrease of crystalline xonotlite (Xon, green points, XRD measurement), and growth of amorphous Co-S-H phase (Co-S-H, orange points, XRD measurement); *y* denotes fractional change. (**b**)   Decrease of Co$^{2+}$ in solution at three initial molal concentrations: b$_1$ 0.35 *m* (red points), b$_2$ 0.073 *m* (grey points), b$_3$ 0.037 *m* (black points); dashed lines are linear fits to data, with best-fit rate constant $k = 0.078\pm 0.006$, $0.072\pm 0.005$ and $0.071\pm 0.001$ mol/(kg CS h) respectively for the three concentrations; mean $0.074\pm 0.004$ mol/(kg CS h).

The only remaining sharp reflection is from minor calcite which does not take part in the sequestration reaction. The xonotlite reflections from the untreated CS are absent in the fully reacted solid. There are two new features not present in xonotlite: a broad asymmetric reflection with a maximum intensity at 34.4$^\circ$ $2\theta$ (*d*-spacing: 0.260 nm), and a broad symmetrical reflection centred at 59.4$^\circ$ $2\theta$ (*d*-spacing: 0.155 nm). These features resemble the 2D (*hk*) diffraction bands first described in disordered layer materials such as graphite^40^ and the sheet silicate halloysite^41^. These broad reflections are also found in Co phyllosilicates^30,39,42,43^ produced in small quantities by co-precipitating aqueous solutions of cobalt chloride CoCl$_2$ with sodium metasilicate Na$_2$SiO$_3$ rather than sequestering Co in a preformed solid material as we do here. Before the time required for full replacement (5 days for $b({\textrm{Co}})_0$ = 0.35 *m*), Co-S-H and xonotlite co-exist as shown in Fig. 3a. When the solution does not contain enough Co to replace all Ca in the xonotlite contained in 1.00 g of CS, as in $b({\textrm{Co}})_0$ = 3.5–73 m*m*, Co-S-H and xonotlite also co-exist in the final product (see Supplementary Information). Quantification of the xonotlite remaining and reaction product formed over time using $b({\textrm{Co}})_0$ = 0.35*m* is shown in Fig. 4a. From the observed *d*-spacings of the (*hk*) bands in the reaction product, we obtain lattice spacings for the silicate sheet $a = 0.535$ nm and $b = 0.930$ nm (with $b \approx \surd {3}a)$, in close agreement with those obtained previously^39^ for the Co phyllosilicates Co$_6$Si$_8$O$_{20}$(OH)$_4$ and Co$_6$Si$_4$O$_{10}$(OH)$_8$. This indicates that the reaction product, although highly disordered, contains intact fragments of silicate sheet structures.

Further confirmation of this comes from FTIR and XAS analyses. FTIR spectra of Co-S-H lack the band at 1197 cm^−1^  assigned to the crosslinks in the double-chain structure of xonotlite^44^, showing that the double chain structure is dismantled. The band at 1068 cm^−1^  broadens to a shoulder on formation of Co-S-H. Further evidence of silicate chain disruption comes from changes to the strong band at 960 cm^−1^  in the unreacted xonotlite (Si–O–Si stretching vibrations of non-bridging tetrahedra^37^). In Co-S-H this is replaced by a strong, broad band at 985 cm^−1^, assigned to the Si-OH stretching mode in silanol groups^45^. The product is more highly hydrated than the starting CS as shown by broad bands in the region 3300–3650 cm^−1^  present in the reaction product but not in CS itself. The feature at 1640 cm^−1^ also present only in the reacted product, is assigned to the H-O-H bending mode of molecular water^37^ (not from OH groups), and confirms that some molecular water is present in the product. Together, these analyses show that the double chain structure of xonotlite has broken down, that the Co-S-H product is hydrated and largely amorphous but has phyllosilicate-like features.

Comparing the shape and edge energy position of the XANES spectra (Fig. 3c and d) of standards and samples show that Co does not oxidise during reaction with xonotlite in the presence of NO${_3}^{-}$, and that Co(OH)$_2$ is not present in the reacted samples. The Co co-ordination environment is similar in Co-S-H produced at all $b({\textrm{Co}})_0$ concentrations.

To understand the Co-S-H structure, EXAFS spectra are fitted with relevant and available structures. As structural information does not exist for hydrated cobalt silicates/phyllosilicates, we chose hydrated silicates of Ni, Mg and Cu as their cation radii (0.83, 0.86, 0.87 Å, respectively) are similar to that of Co (0.89 Å) and the metal ions have 6-fold co-ordination. We chose phyllosilicates and single and double chain silicates to determine if the chain structure of xonotlite is preserved. The models based on chain structures includes the metal-metal and metal-Si scattering paths at interatomic distances (2.6–2.8 Å, and 3.4–3.5 Å) that could not be fitted to the Co EXAFS (see Supplementary Information). The structure of the Co-S-H phase is therefore not similar to these inosilicates. In contrast, all Co EXAFS spectra could be fitted with the models based on the phyllosilicates. These fits include three distinct scattering paths which all statistically improved the respective fits (see Supplementary Information): a Co-O scattering path with a coordination number [CN] of 6 at 2.085 Å, a Co-Co scattering path with a CN of 4.3–4.9 at 3.13 Å, and a Co-Si scattering path with a CN of 3.9–5.6 at 3.31 Å. We conclude that structure of the Co-S-H phase resembles a phyllosilicate, confirming the XRD and FTIR results. Furthermore, the EXAFS results highlight that this phyllosilicate-like Co-S-H phase is the dominant reaction product even at the lowest concentration: $b({\textrm{Co}})_0$ = 0.0035 *m*.

#### Mass change on sequestration

Accurate measurements of sample mass show that considerable water is taken up during Co-S-H formation. In tests on small blocks of CS the mass increased by 46 percent (Table 1) on Co-treatment, of which only 16 percent is accounted for by replacing Ca with Co. Solution data show that all Ca is replaced by Co and that no Si is lost by the solid, so that the remaining mass gain must occur by incorporation of water, either as hydroxyl groups OH or as molecular water H$_2$O or both. The mass of water in Co-S-H (here conditioned at 11 percent RH at 25  $^\circ$C) corresponds to $\approx 13$ mol water per mol of xonotlite in the starting material. We write the reaction stoichiometry as:

1 $$\begin{aligned} {6\hbox {Co}^{2+}} + {\hbox {Ca}_{6}}{\hbox {Si}_{6}}{\hbox {O}_{17}}{(\hbox {OH})_{2}} +{13 {\hbox {H}_{2}}\hbox {O}} \rightarrow {{\hbox {Co}_{6}}{\hbox {Si}_{6} \text{O}_x}}{(\hbox{OH})}_{y}.{z}\,{{\hbox {H}_2}\hbox {O}} + {6\hbox {Ca}^{2+}} \end{aligned}$$

where $x=4+z$ and $y=28-2z$ by charge balance.

**Table 1:** Mass change of CS blocks on complete Co reaction.

Sample	Mass ratio	Mol ratio	Mol ratio	Bulk density	Solid density	Porosity
	$w_a/w_b$	$n_\mathrm{{H_{2}O}}/n_\mathrm{Xon}$	$\Delta n_{{\mathrm{H_{2}}\mathrm{O}}}/n_\mathrm{Xon}$	$\rho _b$ kg/m$^3$	$\rho _s$ kg/m$^3$	*f*
	Col a	Col b	Col c	Col d	Col e	Col f
A	1.483	13.8	3.2	415	2760	0.850
B	1.482	13.8	2.8	–	–	–
C	1.438	11.9	2.8	400	2635	0.849
D	1.452	12.5	3.0	395	2675	0.852
Mean	1.464	13.0	2.9	400	2690	0.850
CS control				270	2540	0.895

(a) Sample weights before reaction ($w_b$), and after reaction ($w_a$). All samples were conditioned over LiCl saturated solution at 25.0$^\circ$C  (RH 11.3 percent^46^) prior to weighing. (b) Mol ratio where $n_\mathrm{{{H_{2}}O}}$ is the water incorporated in the reaction product calculated from weight gain ($w_a-w_b$) after allowing for the mass change associated with replacement of Ca by Co; and $n_{\textrm{Xon}}$ is the xonotlite amount before reaction. (c) $\Delta n_\mathrm{{H_{2}O}}$ is calculated from weight loss on conditioning the reacted samples over molecular sieve 4A desiccant at 25 $^\circ$C  (RH $<0.1$ percent)^47^. (d)–(f) Bulk density, solid density and porosity are calculated using the standard Archimedes buoyancy methods^48^ with water as the suspending and saturating liquid

Samples lose only 20 percent of the incorporated water when conditioned over a molecular sieve 4A desiccant. We regard this as a rough measure of the amount of loosely held molecular water at that RH (< 0.1 percent at 25  $^\circ$C). Therefore most of the incorporated water is chemically combined as OH. Water vapour sorption isotherms show that Co-S-H is hygroscopic and takes up about ten times as much water as CS over the RH range 0–80 percent at 25  $^\circ$C (see Supplementary Information).

From Table 1, we have $z\approx 3$, so that Co-S-H has the empirical formula Co$_6$Si$_6$O$_7$(OH)$_{22}$.3H$_2$O. Equation (1) can be written succinctly as

2 $$\begin{aligned} {6{\hbox {Co}^{2+}}} + {\hbox {Xon}} +{13 {\hbox {H}_{2}}\hbox {O}} \rightarrow {\hbox {Co-S-H}} + {6{\hbox {Ca}^{2+}}}. \end{aligned}$$

The molecular water combines reversibly and is zeolitic and non-structural. There is evidence for the hydrous nature of Co-S-H from the IR bands in the region 3300–3650 cm$^{-1}$  present in Co-S-H but not in CS. The weak feature at 1640 cm$^{-1}$  confirms that a small quantity of molecular water is present in Co-S-H at ambient relative humidity, as we find gravimetrically. The reaction scheme of Eq. (1) implies that the double-chain silicate unit (Si$_{6}$O$_{17}$)$^{-10}$ is dismantled during the reaction. This is supported by the disappearance of the IR band at 1197 cm^−1^, previously assigned to the crosslinks between the double chains of xonotlite. We have shown the structure is poorly ordered (XRD, IR, XAS), hydrated (IR, mass change) and resembles phyllosilicate fragments (XRD, XAS), which we expect to be edge-terminated with OH groups for charge neutrality. We have direct knowledge of the Co-S-H elemental composition from the starting xonotlite, the Ca/Co exchange reaction and the fact that Si is retained, together with the mass changes during Co-S-H formation. This combined with charge balance constraints leads to Eq. (1). To determine *z* we make use of the dehydration mass change ($z\approx 3$), which leads us to Eq. (2) and provides the complete empirical formula.

#### Sequestration kinetics

To determine the kinetics of the sequestration reaction, we use the ICP and quantitative XRD data. We show (Fig. 4a) that in contact with Co solution the release of Ca into solution occurs at a constant rate. The amount of Ca released closely tracks the disappearance of crystalline xonotlite in the CS as determined by quantitative XRD (Fig. 4a), and also tracks the appearance of the Co-S-H phase. This constant rate is maintained down to low Co concentrations and until the reaction ceases abruptly, either because all available Co has been sequestered or until all xonotlite has been consumed. In Fig. 4a, where the initial Co concentration is 0.35 *m*, the timescale for complete release of Ca and complete decomposition of xonotlite is about 105 hours. The total amount of Ca released into solution is $7.60 \pm 0.30$ mmol, and the amount of xonotlite initially present in the solid CS is 1.30 mmol. This mol ratio (7.60/1.30) = $5.8 \pm 0.2$ is very close to the expected stoichiometric ratio of 6.

How the amount of Co in solution decreases with time is a direct measure of the rate of Co sequestration. Figure 4b shows this for three different initial Co concentrations. Remarkably, the rate is the same for all: which means that the rate of sequestration is independent of the initial Co concentration over a tenfold variation in that concentration, and remains independent of Co concentration to the lowest measured levels. The reaction therefore has zero-order kinetics with respect to Co. The Co sequestration rate is controlled by the rate of release of Ca resulting from the dissolution or decomposition of xonotlite. Several such zero-order dissolution-controlled reactions are known in rock-fluid systems^49^. For Co sequestration we write

3 $$\begin{aligned} {\mathrm d}n_\mathrm{Co}/{\mathrm d}t= -m_\mathrm{CS} k, \end{aligned}$$

or

4 $$\begin{aligned} n_\mathrm{Co}(t)=n_\mathrm{Co}(0)-m_\mathrm{CS}kt, \end{aligned}$$

where $n_\mathrm{Co}$ is amount of Co (mol) in solution, $m_\mathrm{CS}$ the mass of CS sequestrant used, and *k* the rate constant. In these tests, the best-fit rate constant $k_0=0.074\pm 0.004$ mol/(kg CS h) at 25 $^\circ$C. In practical terms, this corresponds to a rate of removal in a well-mixed reactor of 105 kg Co per tonne of CS per day. It is likely that the quantity $m_\mathrm{CS}$ is a proxy for the Si content of the solid phase, which is conserved (and therefore constant) throughout the sequestration process. The amount of Co-S-H formed is in direct proportion to the amount of Si present in the weight of CS sequestrant used.

The time for the complete removal of Co from solution is proportional to the initial amount of Co in solution, irrespective of concentration, as is diagnostic of a zero-order reaction. These values are $103\pm 6$, $23\pm 1$, $12\pm 2$ and $0.8\pm 0.2$ hours for equal volumes of solution at the four initial concentrations $b({\textrm{Co}})_0$ 0.350, 0.073, 0.037 and 0.0035 *m*, each in contact with 1.00 g CS. Generally, the time for Co removal in these tests is $0.29\,(b({\textrm{Co}})_0/{\mathrm m}m)$ h. We expect that the rate constant depends on the particle size of the CS, and of course on temperature.

Further confirmation of the sequestration rate constant comes from filter-column experiments (see Supplementary Information), in which a Co solution percolates through a CS bed at a controlled flow rate. Combined results show that CS sequesters Co$^{2+}$ from aqueous solution at a constant rate, independent of the Co concentration, up to a maximum capacity of about 7.5 mol Co per kg CS, or about 440 g Co per kg CS.

#### Selectivity

We note that relatively few other elements are present in the natural environment as divalent metal cations at high solution concentrations. The results reported here show that Ca$^{2+}$ does not interfere with Co$^{2+}$ sequestration. Scoping experiments show that high concentrations of Mg$^{2+}$ do not interfere, and neither do the monovalent ions Na$^+$ and K$^+$. We have extensive data to show that Ni$^{2+}$ behaves in much the same way as Co$^{2+}$, and that both ions are sequestered simultaneously from mixed Co$^{2+}$/Ni$^{2+}$ solutions (to be published).

### Sharp front sequestration transport model

**Figure 5:**
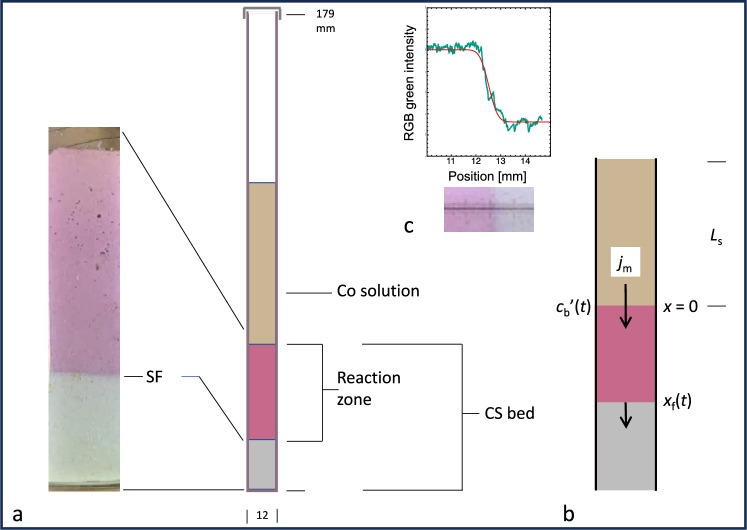
Sharp front experiment and model. (**a**)   Photograph (left hand side) of a reacted sample after penetration of Co$^{2+}$ into a CS bed at 25  $^\circ$C, showing the well-defined reaction zone (pink) and reaction front at $x=x_\mathrm{f}$. The initial Co(NO$_3$)$_2$ concentration = 0.33 *m* and CS bed volume fraction porosity $f_\mathrm{b}$ = 0.91. The sample was water saturated to avoid capillary transport of Co$^{2+}$. The image was taken at $t=$ 894 hours and the reaction front position $x_\mathrm{f}$ is 16.4 mm below bed surface. (**b**)   Schematic of Sharp Front model (see text): $x_\mathrm{f}(t)$ is the position of the reaction front at time = t, $L_\mathrm{s}$ the length of the solution-filled part of the tube, $c_\mathrm{b}^\prime (t)$ the Co concentration at the bed surface and $j_{\mathrm m}$ the Co mass flux across the bed surface. (**c **) Photograph (bottom) showing how the location of the reaction front is determined from analysis of the green channel image intensity (top), here 12.51 mm at the midpoint of the ${\text {erfc}}(x)$ fit (—), measured from the bed surface.

When a Co solution is brought into contact with a sedimented powder bed or a block of CS the progress of the sequestration is visible as a moving reaction front. In the experimental arrangement of Fig. 5a, a Co(NO$_3$)$_2$ solution containing a total initial mass $m_{\mathrm s0}$ of Co is in contact with the upper surface of a water-saturated CS bed of volume fraction porosity $f_\mathrm{b}$. The reaction front is steep and its position, $x_{\mathrm f}$, well defined: this prompts the use of a Sharp Front [SF] model. SF models have been used to describe water transport by capillary flow through porous materials^50,51^, but a new SF model is developed here to describe a transport process that incorporates the chemical sequestration.

The SF test directly simulates Co removal from contaminated waters by blocks of CS, as loose powder would be more difficult to deploy and retrieve during environmental remediation. The SF model is highly successful in describing the movement of the reaction front therefore it should be widely applicable in modelling reaction zones in industrial and environmental mineral systems.

Given that the SF system in Fig. 5b is closed, the total mass of Co is constant and so $m_{\mathrm s}+m_{\mathrm r}=m_{\mathrm s0}$, with $m_{\mathrm s}$ the total mass in solution, and $m_{\mathrm r}$ the total mass immobilized in the reaction zone. The solution mass concentration $c_{\mathrm s}^\prime = m_{\mathrm s}/(A L_{\mathrm s})$ where $L_{\mathrm s}$ is the length occupied by the solution in the tube of cross-section area *A*. Then the Co mass flux across the upper surface of the bed at $x=0$ is $j_{\mathrm m} = A^{-1}\cdot {\mathrm d} m_{\mathrm r}/{{\mathrm d}t}$. We write the simple linear transport relations

5 $$\begin{aligned} \frac{{\mathrm d} m_{\mathrm r}}{{\mathrm d}t} = AK\frac{c_{\mathrm b}^\prime }{x_{\mathrm f}} = \frac{K}{\gamma L_{\mathrm s}} \frac{m_{\mathrm s0}-m_{\mathrm r}}{m_{\mathrm r}}, \end{aligned}$$

where $x_{\mathrm f} = \gamma m_{\mathrm r}$, with $\gamma$ the length of the reaction zone per unit mass of sequestered Co, and *K* is a transport coefficient, dimension ${{\textsf{L}}^{2}} {\mathsf T^{-1}}$ (length squared/time, same as diffusivity). We assume that the solution is well mixed, and that the Co concentration at the bed surface, $c_\mathrm{b}^\prime$, is the same as the solution concentration, $c_\mathrm{s}^\prime$. The mass concentration of Co, $c^\prime =c M_\mathrm{Co}=b \rho _\mathrm{soln}/(1+b M_\mathrm{Co})$, where *c* is the amount concentration (mol Co/m$^3$), *b* the molality (mol Co/kgw), $M_\mathrm{Co}$ the molar mass of Co, and $\rho _\mathrm{soln}$ the solution density.

In terms of the experimental variable $x_{\mathrm f}$, Eq. (5) becomes

6 $$\begin{aligned} \frac{{\mathrm d}x_{\mathrm f}}{{\mathrm d}t} =\frac{K}{L_{\mathrm s}}\cdot \bigg [\frac{x_{\mathrm f\infty }}{x_{\mathrm f}}-1\bigg ], \end{aligned}$$

where $x_{\mathrm f\infty }=\gamma m_{\mathrm s0}$ is the final (equilibrium) position of the reaction front when all Co has accumulated in the reaction zone ($m_{\mathrm s}=0$). The advance of the front is eventually halted by the removal of Co from solution. Integrating Eq. (6) with initial condition $x_{\mathrm f}=0$ at $t=0$ gives

7 $$\begin{aligned} -x_{\mathrm f} - x_{\mathrm f\infty } \ln \bigg [1-\frac{x_{\mathrm f}}{x_{\mathrm f\infty }}\bigg ] = \frac{K}{L_{\mathrm s}} t. \end{aligned}$$

At early times $x_{\mathrm f} = (2K x_{\mathrm f\infty }/L_{\mathrm s})^{1/2}\cdot t^{1/2}$. The non-dimensional form of Eq. (7) with $X=x_{\mathrm f}/x_{\mathrm f\infty }$, and $T= K t/(x_{\mathrm f\infty } L_{\mathrm s})$ is

8 $$\begin{aligned} -X - \ln (1-X)=T, \end{aligned}$$

with $X=(2T)^{1/2}$ at early times.

**Figure 6:**
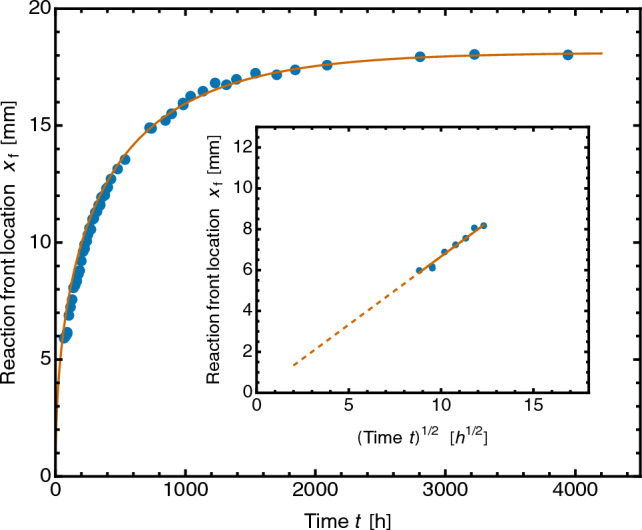
Position of the Co sequestration reaction front $x_\mathrm{f}(t)$: experimental data (circles) fitted to the Sharp Front model (solid line), Eq. (7): $t= \alpha _0[-x_\mathrm{f}-\alpha _1\ln (1-x_\mathrm{f}/\alpha _1)]$, with best-fit parameters $\alpha _0 = 44.53\pm 1.17$mm^−1^ h, $\alpha _1= 18.13\pm 0.04$ mm. In this test, $b({\textrm{Co}})_0 = 0.331$ *m*, $L_{\mathrm s}=73.5$ mm, $\rho _\mathrm{b}=203.5$ kg/m$^3$ and $A=90.1$mm^2^. Inset shows that $x_{\mathrm f}$ varies as $t^{1/2}$ at early time, with the best-fit slope = $0.658\pm 0.003$ mm h$^{-1/2}$.

In experimental tests, the position $x_{\mathrm f}$ of the reaction front was measured by image analysis as described in Methods below. Figure 6 shows that the SF model describes the motion of the reaction front well. There are only two disposable fit parameters, $\alpha _0$ and $\alpha _1$. In fact, $\alpha _1= x_{\mathrm f \infty }$, the final location of the reaction front, and this is obtained by direct measurement with little error at the end of the experimental run. Since $L_{\mathrm s}$ is known, *K* can be determined immediately from the fit parameter $\alpha _0=L_{\mathrm s}/K$, and from the data of this test is found to be $(4.58\pm 0.10)\times 10^{-10}$ m$^2$ s$^{-1}$.

**Figure 7:**
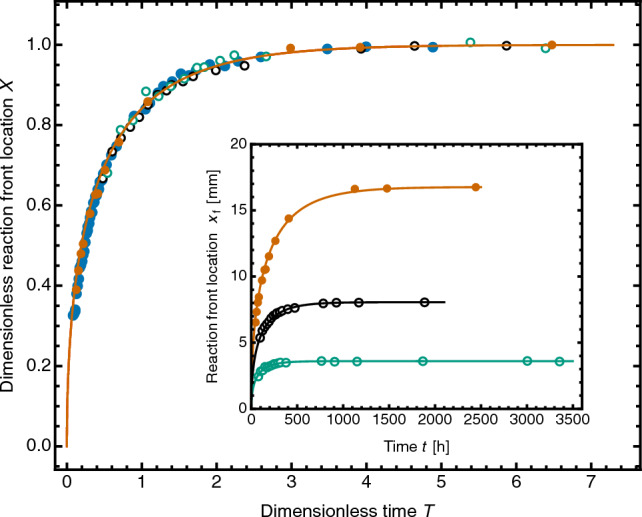
Advance of the Co sequestration front in CS: four experimental datasets scaled to the dimensionless form of the SF model, Eq. (8) (−). Data as Fig. 6 for blue filled circles; orange filled circles, $b({\textrm{Co}})_0 = 0.331$*m*, $L_{\mathrm s}=47.8$ mm, $\rho _\mathrm{b}=139.9$ kg/m$^3$; black open circles, $b({\textrm{Co}})_0 = 0.331$ *m*, $L_{\mathrm s}=36.9$ mm, $\rho _\mathrm{b}=221.4$ kg/m$^3$; green open circles, $b({\textrm{Co}})_0 = 0.161$ *m*, $L_{\mathrm s}=38.9$ mm, $\rho _\mathrm{b}=273.6$ kg/m$^3$. Inset shows the unscaled experimental data ($x_{\mathrm f},t$) fitted to Eq. (7). For fit parameters, blue filled circles, see Fig. 6; orange filled circles, $\alpha _0= 22.52\pm 0.96$ mm^−1^ h, $\alpha _1=16.82\pm 0.08$ mm; black open circles, $\alpha _0=26.01\pm 0.80$ mm^−1^h, $\alpha _1=8.04\pm 0.05$ mm; green open circles, $\alpha _0=39.50\pm 2.87$  mm^−1^h, $\alpha _1=3.60\pm 0.0.04$ mm.

Figure 7 shows data from four tests in which the volume and molality of the Co solution were varied, as was the packing density of the CS bed. The scaled data (*X*, *T*) are described well by the dimensionless form of the SF model, Eq. (8), which therefore provides the master curve for the sequestration kinetics.

An important quantity derived from the fit parameter $\alpha _1$ is the Co:Ca stoichiometry of the reaction. Since $\alpha _1 = x_{\mathrm f \infty }$, then $\gamma =\alpha _1/m_{\mathrm s0}$, the ratio of the final length of the reaction zone to the initial mass of Co in solution. The packing density of the bed $\rho _\mathrm{b}$ is known, and $1/(A\gamma \rho _\mathrm{b})$ is the mass of Co sequestered per unit mass of CS.

**Figure 8:**
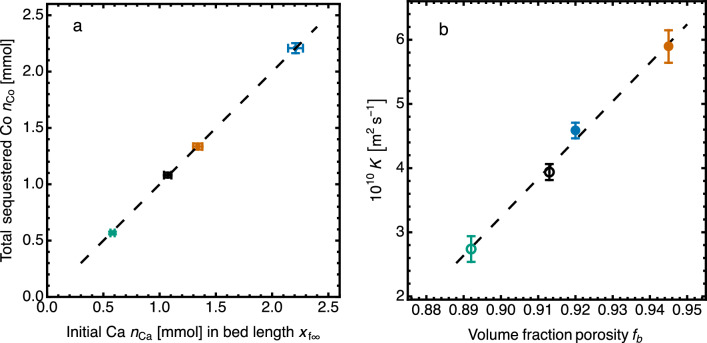
Sharp Front analysis of four experimental tests of Co sequestration by replacement of Ca in CS beds (symbols key as Fig. 7; uncertainties shown graphically). (**a**): Replacement of Ca by Co at equilibrium, dashed line $n_\mathrm{Co} = (1.00 \pm 0.01)n_\mathrm{Ca}$. (**b**) Transport parameter *K*, showing increase of *K* with bed porosity $f_\mathrm{b}$.

We then compare the total amount (mol) of Co sequestered with the amount of xonotlite Ca originally present in the length $\alpha _1=x_{\mathrm f\infty }$, the equilibrium location of the reaction front. Figure 8a shows that this mol ratio is 1.00$\pm 0.01$, demonstrating that all the Ca available in the CS in the reaction zone is replaced by Co. The Ca/Co replacement is stoichiometric and complete, confirming the same result from solutions analysis of a stirred system using powdered CS. Figure 8b shows also that the transport parameter *K* is sensitive to the bed porosity *f*. The extrapolated value of *K* at $f=1$, $\approx 9.2\times 10^{-10}$ m$^2$/s, is close to reported values of the diffusivity of Co$^{2+}$ ion in water at similar concentrations^52,53^. This shows that Co sequestration in the porous solid is controlled by the slow Co$^{2+}$ ion diffusion in the bed, not by the much faster kinetics of the sequestration reaction measured in the stirred-batch experiments using CS powder. In cases such as this, the SF experiment provides a simple way to determine accurately the diffusivity of the metal ion in a porous material.

### Co-CS reaction as a dissolution/precipitation replacement

Changes in the mineral composition of rocks are often mediated by water through coupled dissolution and precipitation [CDP]. The recent concept of CDP replacement^32^ has wide impact in geological mineralogy. The Co-CS sequestration reaction we describe is the first example of CDP replacement in a synthesized industrial material. The sequestration reaction has three essential CDP characteristics: reactant and reaction product are close-coupled spatially; the reaction front is sharp rather than diffuse; and the reaction product is permeable to allow the continuing reaction. The Co-CS reaction is novel in producing an amorphous rather than a crystalline product. Our quantitative SF transport model is applicable to geological CDP replacement where transport modelling is so far absent.

### Conclusions

Here we break new ground by demonstrating the total sequestration of Co$^{2+}$ from concentrated solutions by a commercial calcium silicate hydrate material with exceptionally high sequestration capacity. The sequestration occurs spontaneously at ambient temperature, removes 0.44 kg of Co per 1 kg of CS in under 5 d and is a replacement reaction that does not reverse when the Co-S-H product is in contact with aqueous Ca after its formation. Other materials that can remove Co from aqueous solution are at least $\times$5 less effective and do so by surface or interlayer sorption^14^.

These results demonstrate that CS is a fast, easy and economic sequestrant for removing Co from solution, particularly for environmental and nuclear $^{60}$Co clean-up targets. Importantly CS could be deployed in large blocks rather than powders, as SF tests show. The reaction product Co-S-H is X-ray amorphous and has a large surface area. The silicate double chains of CS are destroyed in the sequestration which occurs by coupled dissolution-precipitation. There is evidence that Co-S-H contains fragments of phyllosilicate structures. Amorphous Co-phyllosilicates are known to act as water-splitting catalysts^43^, replacing expensive precious-metal catalysts in producing hydrogen. Sequestration kinetics are zero-order in Co concentration, so that the rate is maintained even at low Co concentrations. The reaction goes to completion leaving at most only ppm residual Co.

A new SF experiment design and analysis give access to chemical and transport parameters where Co solution is in direct contact with the solid sequestrant. SF methodology has wide applicability in fundamental materials studies, in process and environmental engineering. The SF and filter-column experiments provide the data required for applications scale-up.

## Methods

### Materials

The sequestrant used is Calsitherm insulation board (Calsitherm Silikatbaustoffe GmbH, Paderborn), a commercial product manufactured from lime and silica by steam autoclaving at 150–200  $^\circ$C  for 15–25 h^54^.

### Analysis of solutions

Solution compositions are reported as molality *b* (unit mol/kg water, denoted mol/kgw or *m*). In transport models we use the amount concentration *c* (mol/m$^3$) and the mass concentration $c^\prime =c M_\mathrm{A}$ (kg A/m$^3$), where $M_\mathrm{A}$ is the molar mass of substance A. Samples were prepared by adding 25 mL of Co(NO$_3$)$_2$ solution (concentration range 0.35 *m* to 0.0035 *m*) to 1.00 g of CS in 50 mL centrifuge tubes, and agitated on a shaker tray for 10 min up to 56 days. Each supernatant was separated by centrifuging, the liquid phase acidified and diluted with 3 percent HNO$_3$ and divided into triplicate samples for ICP-OES analyses. The solid was washed in DI water, centrifuged and dried to constant weight at 40  $^\circ$C. The slurry pH was measured before centrifuging.

### Characterisation of solids

#### Total carbon [TC] and total organic carbon [TOC] analyses

TC and TOC analyses were carried out by Elemental Microanalysis Ltd (Devon, UK). For TC, the dried samples (40  $^\circ$C) were weighed into silver capsules, placed in a combustion tube (1000  $^\circ$C) and burned in pure oxygen. Combustion gases were passed over catalysts to ensure complete oxidation and absorption of halogens, sulphur and other interferences. CO_2_ was separated on a chromatographic column and quantified using a thermal conductivity detector. TOC analyses were carried out in the same way on samples pre-treated with 15 M HCl to remove carbonates.

#### X-ray fluorescence [XRF]

XRF analyses were carried out by AMG Analytical Services Ltd. Analysis was carried out on powdered samples fused into glass beads with Li$_2$B$_4$O$_7$ at 1270 $^\circ$C  and on six certified reference materials using a PANalytical AXIOS wavelength-dispersive instrument.

#### X-ray diffraction [XRD]

XRD patterns were collected using a PANalytical Empyrean system in reflection mode (Bragg-Brentano$^\textsf{HD}$ module) with a Cu anode (45 kV and 40 mA). The detector PHD lower level was set to 50 percent, optimised to suppress fluorescence from the Co-containing samples. Slit and Soller settings were chosen to avoid beam spill on the sample (dia 16 mm). Primary (14 mm) and secondary (6 mm) masks and a fixed divergence slit of 0.125$^\circ$ were used on the incident beam side with a 0.03 rad Soller (iCore); and 0.125$^\circ$ anti-scatter slit plus 0.04 rad Soller (dCore) on the divergent beam side. Data were collected on powdered samples in back-loading holders from 5 to 80$^\circ$ $2\theta$, step size 0.0263$^\circ$ $2\theta$, count time 2 s per step, samples spinning at 2 rev/s. Quantification was based on a Partial-Or-No-Known-Crystal-Structure [PONKCS] method^55,56^. As the ICSD structure files do not match exactly the hydrothermal xonotlite in CS, an ‘*hkl* phase’  with PONKCS style cell mass was developed from calibration mixes with CaF$_2$ (Sigma-Aldrich, > 99.9 percent) as a standard. A similar approach was used for the amorphous Co-S-H. TOPAS v5 and EVA v5.2 (Bruker Ltd) were used for data analysis and phase identification.

#### Fourier transform infrared spectroscopy

Powdered samples were analysed using an Agilent 4500 ATR FTIR spectrometer. Each sample was run with 64 scans at a resolution of 4cm^−1^  .

#### X-ray absorption spectroscopy XAS

Spectra were obtained from solid samples collected from all $b({\textrm{Co}})_0$ concentrations after 56 days and from Co$^{2+}$ (aqueous), $\beta$-Co(OH)$_2$ and Co$_3$O$_4$ standards. Dried solids were diluted with BN and pressed into a 100–400 $\upmu$m thick pellets for analysis at beamline B18 at Diamond Light Source. The Co K-edge (7.709 keV) was measured using a Si(111) monochromator. Samples $b({\textrm{Co}})_0$ = 0.35 *m* and 73  m*m*, Co(OH)$_2$ and Co$_3$O$_4$ were analysed in transmission mode, and samples $b({\textrm{Co}})_0$ = 0.037 *m* and 0.0035 *m*, and aqueous Co$^{2+}$ in fluorescence mode with a 36-element Ge detector. Spectra were processed with Demeter software package. Athena was used for data reduction and background subtraction and Artemis to fit the *R*-space Fourier transform of the EXAFS spectra^57^.

### Reaction front analysis

The positions of the reaction front $x_\mathrm{f}$ were obtained from images of the sedimented CS bed. The position of the bed surface did not change during the reaction, within the precision of the measurement, $\approx \pm \,\, 0.02$ mm. The green-channel intensity had the greatest RGB contrast and was extracted from the image pixel by pixel along the bed ($\approx 4.6\,\, \upmu$m pixel^−1^). The front location $x_{\mathrm f}$ was taken as the midpoint of an error-function fit (Fig. 5c), with typical standard uncertainty 0.013 mm. For fitting $x_\mathrm{f}(t)$ data, Eq. (7) may be written as

9 $$\begin{aligned} t= \alpha _0 \left[ -x_\mathrm{f}-\alpha _1\ln (1-x_\mathrm{f}/\alpha _1)\right] \end{aligned}$$

with regression parameters $\alpha _0=L_\mathrm{s}/K$ and $\alpha _1=x_{\mathrm f \infty }$.

### Errors and uncertainties

Errors in measured quantities are either $k=2$ expanded uncertainties estimated from standard deviations of replicates or estimates based on known accuracy of laboratory measurements. Errors in parameters obtained by least-squares regression are $k=2$ uncertainties calculated from standard errors of regression with 0.95 confidence interval. Errors in derived quantities are then calculated by standard methods of error propagation.

### Supplementary Information

Supplementary Information.

## Data Availability

The datasets used and/or analysed during the current study are available from the corresponding author on reasonable request.
