# Supplementary material for: Clean-up of divalent cobalt ions by massive sequestration in a low-cost calcium silicate hydrate material
Source: Sci Rep. 2024 Mar 25;14:7052. doi: 10.1038/s41598-024-56617-x (PMC11336121; doi:10.1038/s41598-024-56617-x)
Supplement: Supplementary file 1 — Supplementary Information. [file 41598_2024_56617_MOESM1_ESM.pdf]

Clean-up of divalent cobalt ions by massive sequestration in a low-cost calcium silicate hydrate material

Andrea Hamilton, Pieter Bots, Han Zhou, Bao Liu, Christopher Hall

Supplementary information

S1 Experimental notes

Materials

We used CS from two batches: CS-1, material from the Hamstad project[1] (bulk density ρ_b 270 kg m^{-3}); and recently purchased material CS-2 (ρ_b 185 kg m^{-3}). XRF and XRD analyses show the mineral compositions of the CS samples are similar: CS-1, 94 wt percent xonotlite, 6 wt percent calcite; and CS-2 89 wt percent xonotlite, 11 wt percent calcite. TC and TOC analyses show both samples contain cellulose fibres: CS-1 (2.4 wt percent cellulose) and CS-2 (3.6 wt percent cellulose). Physical and mineralogical properties of CS have been reported previously[1–5]. In stirred-batch and SF tests the chemical behaviour of CS-1 and CS-2 was indistinguishable.

SF reaction front analysis

To ensure that x_f is the dependent variable in obtaining least-squares estimates of α_0 and α_1 we use as nonlinear fit model the inverse of Equation 7 of

the main paper

$$x_{\mathrm{f}} = \alpha_0 \big[1 + W[-\exp(-1 - t/(\alpha_0 \alpha_1))]\big], \tag{S1}$$

where W is the principal branch of the Lambert W function.

We note also that Equation 8 of the main paper is the same function as the solution to the Lucas-Washburn capillary-rise problem[6] (for example of water in walls), in which the upward advance is eventually halted by gravity.

S2 Characterisation of CS material

Oxide analysis by XRF

Table S1 X-ray fluorescence analysis of CS-2. Oxide is reported as wt percent, total mass closure is 99.52 wt percent. Loss on ignition is reported for 450 °C and 1000 °C and carried out on a separate CS sample.

Oxide or loss on ignition (LOI)	Wt percent
Na_2O	< 0.05
MgO	0.51
Al_2O_3	0.16
SiO_2	45.69
P_2O_5	< 0.05
K_2SO_4	0.05
CaO	45.60
TiO_2	<0.05
Mn_3O_4	<0.05
V_2O_5	<0.05
Cr_2O_3	<0.05
Fe_2O_3	0.10
BaO	<0.05
ZrO_2	<0.05
ZnO	<0.05
SrO	<0.05
LOI (450 °C)	2.86
LOI (1000 °C)	7.74

Total carbon analysis

Table S2 Carbon analysis of CS-2. Three sub-samples were analysed, the average result is reported in the main text. Total carbon was analysed by sample combustion followed by carbon gas separation on a chromatography column with a thermal conductivity detector. For organic carbon analysis, separate samples were acidified by hydrochloric acid digestion at 80°C to remove inorganic carbonates. The only inorganic carbonate present in CS-2 by XRD analysis is calcite. Calcite quantity (6.33 wt percent) is calculated from TC-TOC and cellulose (2.40 wt percent) calculated from TOC.

Carbon analysis	wt percent
Total Carbon	1.87, 1.81, 1.80
Total Organic Carbon	1.06, 1.07, 1.07

Microstructure of CS by SEM

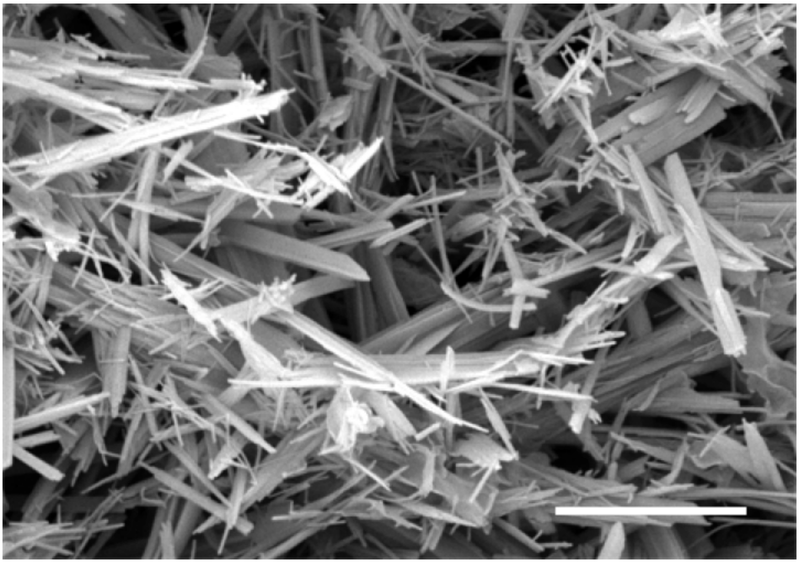

Figure S1 Scanning electron microscope image of CS-1 material captured using secondary electron detector on carbon-coated unpolished sample. Scale bar 2 μm.

Crystallography of xonotlite

The general features of the crystal structure are well established, but complexity arises from the existence of a number of polytypes[7,8]. The polytypes describe the several ordered and disordered stacking arrangements that are possible between chains and sheets. Highly crystalline natural specimens of xonotlite can usually be described as mixtures of distinct ordered polytypes[8–11] but this is not feasible for synthetic xonotlites of poorer crystallinity. None of the X-ray diffraction [XRD] reflections of CS shown in Figure 1a of main paper are unique to individual polytypes.

S3 pH variation during sequestration

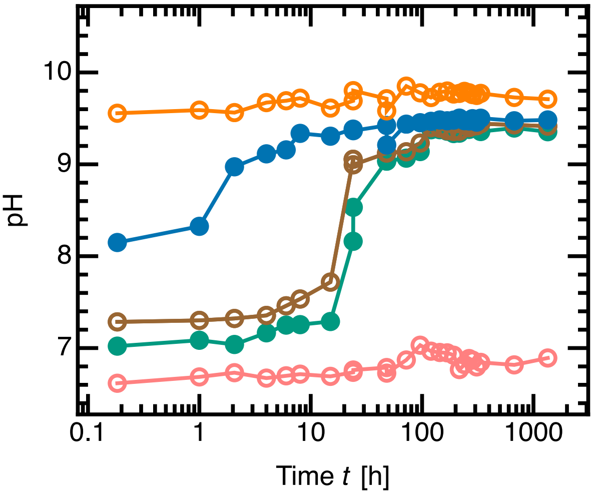

Figure S2 Variation of pH in Co-CS slurries during sequestration at 25 °C; initial $Co(NO_3)_2$ concentration $b(\mathrm{Co})_0$, 0.35 m (pink), 0.073 m (green), 0.037 m (brown), 0.0035 m (blue) and 0 m (orange), decreases upwards from the bottom.

Figure S2 shows the evolution of pH during the sequestration reaction. In the control sample with no Co the pH quickly settles at ≈ 9.7 as a result of the dissolution of xonotlite, a basic and sparingly soluble phase[12,13]. In samples containing Co the pH is lower at early times. This reflects the fact

that $Co(NO_3)_2$ solutions are weakly acidic as a result of slight hydrolysis of Co^{2+}. The initial pH decreases with increasing initial Co concentration. Co-S-H formed in the sequestration of Co has low solubility. In the samples with initial Co concentration 3.5–73 mm the pH rises sharply when the Co is completely sequestered, and ultimately stabilises at ≈ 9.5. These samples all contain residual xonotlite. In contrast, the pH of the sample with initial concentration 0.35 m remains around 6.8 when the reaction is complete because in this case there is no xonotlite and a residual Co concentration of ≈ 23 mm.

S4 X-ray absorption spectroscopy of Co-S-H

X-ray absorption spectra (Co K-edge = 7.709 keV) were collected on standards (Co^{2+} (aq), $Co(OH)_2$ and Co_3O_4) and reacted samples. Data were analysed using two different approaches to fit to available structural data and to determine Co coordination environment. A dual fitting strategy was used to characterise the Co species in the sample. A simple shell-by-shell approach with fixed coordination numbers was used to determine the overall Co speciation and to determine variations in speciation at different initial Co concentrations. The shell-by-shell results were then used to fit the spectra of all experimental samples simultaneously to determine the coordination numbers more accurately[14]. FEFF6[15] was used to calculate the theoretical (initial) scattering paths from the crystallographic information files of a theoretical Co-replaced willemseite ($Ni_3Si_4O_{10}(OH)_2$)[16], β-$Co(OH)_2$[17] and Co_3O_4[18]. The final EXAFS fits are shown as dashed black lines in Figure S3b, c and summarised in Table S4 (standards) and Table S5 (samples).

XANES (X-ray Absorption Near Edge Structure) of the samples (Figure S3a) show a split white line (dash-dot vertical line), and two features at ≈ 7.74 and ≈ 7.77 keV (dashed vertical lines). This is distinctly different

from the Co^{2+}(aq) and Co_3O_4 standards. Due to the presence of trivalent Co in the structure of Co_3O_4, the XANES and white line are shifted to higher energies compared to the other standards and samples. This proves that Co was not in a coordination environment similar to aqueous Co and that Co did not oxidise during its reaction with xonotlite in the presence of NO_3^-. The white line of the $Co(OH)_2$ XANES standard does not show the same split peak as the samples spectra and the two features at $\approx$ 7.74 and $\approx$ 7.77 keV are shifted to lower energies in the $Co(OH)_2$ standard compared to the samples, confirming that $Co(OH)_2$ did not form in the samples.

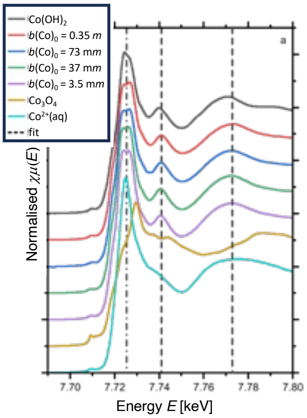

Figure S3 X-ray absorption spectra of the standards (Co^{2+}(aq), β-$Co(OH)_2$ and Co_3O_4) and the Co-S-H-containing samples formed after 56 days in contact with $Co(NO_3)_2$ solutions of concentrations $b(\mathrm{Co})_0 = 0.0035$–$0.35\,m$. Here $\chi\mu(E)$ is the normalised XANES, and $|\chi(R)|$ is the Fourier transform of the k^3-weighted EXAFS $\chi(k)$, where k is the wavenumber.

Table S3 Crystallographic information for β-$Co(OH)_2$, Co_3O_4, willemseite, vermiculite, sepiolite, antophyllite and shattuckite [16,17,19–23]. *Asterisk indicates that due to the complexity and multitude of distinct Mg and Cu crystallographic sites, the average coordination environments of Mg and Cu are simplified.

Phase	Bond	Co-ordination number	Radius (Å)
	Co–O	6	2.12
$Co(OH)_2$	Co–Co	6	3.19
	Co–O	5.33	1.91–1.94
Co_3O_4	Co–Co	4	2.85
	Co–Co	8	3.35
Willemseite	Ni–O	6	2.07–2.08
$Ni_3Si_4O_{10}(OH)_2$	Ni–Ni	6	3.05–3.07
	Ni–Si	4	3.19–3.21
Mg-Vermiculite	Mg–O	1	1.83
$Mg_6Si_8O_{20}(OH)_4 \cdot 4\,H_2O$	Mg–O	5	2.01–2.14
	Mg–Mg	6	3.08–3.09
	Mg–Si	4	3.24–3.26
Sepiolite	Mg–O	6	2.00–2.25
$Mg_4Si_6O_{15}(OH)_4 \cdot 6\,H_2O$	Mg–Mg	5	3.00–3.04
	Mg–Mg	4	3.24–3.32
Antophyllite*	Mg–O	4	1.93–2.18
$Mg_7Si_8O_{22}(OH)_2$	Mg–O	2	2.28–2.41
	Mg–Mg	3	2.87–3.05
	Mg–Si	1	3.04–3.18
	Mg–Mg	2	3.14–3.23
	Mg–Si	1	3.28–3.33
	Mg–Mg	1	3.41
	Mg–Si	3	3.42–3.49
Shattuckite*	Cu–O	4	1.89–2.06
$Cu_5Si_4O_{12}(OH)_2$	Cu–O	2	2.59–2.78
	Cu–Cu	1	2.69
	Cu–Cu	2	3.03–3.13
	Cu–Si	2	2.93–3.18
	Cu–Cu	2	3.32–3.39
	Cu–Si	1	3.50–3.68

Table S4 Summary of the fits for the standards using the second fitting method (see Methods): Co^{2+}(aq), β-$Co(OH)_2$ andCo_3O_4. Listed are the k- and R-range used for fitting the EXAFS spectra, the R-factor for the best fit, the energy shift ΔE_0, the coordination number, radial distance (R) and the Debye-Waller factor (σ^2) of the respective scattering paths. The numbers in parentheses are the uncertainties of the last digit(s) calculated by ARTEMIS[24]. *Asterisks denote values that were fixed during fitting.

Sample	Co^{2+}(aq)	β-$Co(OH)_2$	Co_3O_4
k-range (Å^{-1})	3–12.5	3–12.5	3–12.5
R-range (Å)	1–2.5	1–3.5	1–3.5
R-factor	0.0106	0.0130	0.0073
ΔE_0 (keV)	$-1.3(8)$	$-1.7(8)$	$-0.7(7)$
Scattering path	Co−O	Co−O	Co−O
Co-ordination number	6*	6*	5.333*
R (Å)	2.083(8)	2.101(8)	1.914(6)
σ^2 (Å)	0.007(1)	0.008(1)	0.0031(9)
Scattering path		Co−Co	Co−Co
Co-ordination number		6*	4*
R (Å)		3.175(6)	2.856(6)
σ^2 (Å)		0.0067(7)	0.0028(6)
Scattering path			Co−Co
Co-ordination number			8*
R (Å)			3.361(8)
σ^2 (Å)			0.0057(8)

Table S5 Summary of the fits for the Co-S-H samples using the second fitting method (see Methods). Listed are the k- and R-range used for fitting the EXAFS spectra, the R-factor for the best fit, the energy shift (ΔE0), the coordination number, radial distance (R) and the Debye-Waller factor (σ^2) of the respective scattering paths. In parentheses are the uncertainties of the last digit(s) calculated by ARTEMIS[24]. *Asterisks denote values that were fixed during fitting. **Double asterisks denote values that were fitted to the same value for each sample.

	Initial concentration $b(\mathrm{Co})_0$			
Sample	0.35 m	73 mm	37 mm	3.5 mm
k-range (Å^{-1})	3–12.5	3–12.5	3–12.5	3–12.5
R-range (Å)	1.3–3.5	1.3–3.5	1.3–3.5	1.3–3.5
R-factor	0.0072	0.0072	0.0072	0.0072
ΔE_0 (keV)	$-2.9(8)$	$-3.7(5)$	$-3.6(7)$	$-2.8(5)$
Scattering path	Co−O	Co−O	Co−O	Co−O
Co-ordination number	6*	6*	6*	6*
R (Å)**	2.085(4)	2.085(4)	2.085(4)	2.085(4)
σ^2 (Å)**	0.0073(7)	0.0073(7)	0.0073(7)	0.0073(7)
Scattering path	Co−Co	Co−Co	Co−Co	Co−Co
Co-ordination number	4.9(20)	4.6(20)	4.6(18)	4.3(18)
R (Å)**	3.13(1)	3.13(1)	3.13(1)	3.13(1)
σ^2 (Å)**	0.008(3)	0.008(3)	0.008(3)	0.008(3)
Scattering path	Co−Si	Co−Si	Co−Si	Co−Si
Co-ordination number	5.6(11)	5.3(15)	4.8(10)	3.9(16)
R (Å)**	3.31(3)	3.31(3)	3.31(3)	3.31(3)
σ^2 (Å)**	0.008(3)	0.008(3)	0.008(3)	0.008(3)

S5 Thermal analysis of Co-S-H

Thermogravimetric analysis (TGA) of the reaction product Co-S-H (Figure S4) shows that there is a fractional weight loss of 0.04 on heating from

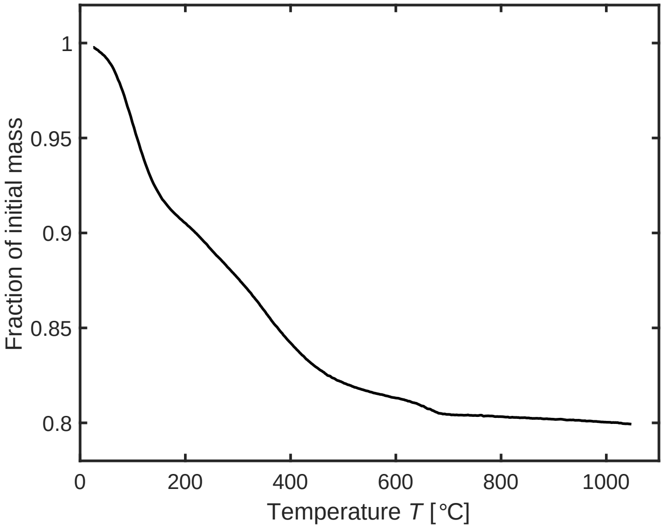

Figure S4 Thermogravimetric trace of Co-S-H, Netzsch STA 449 f3 thermal analyzer, heating rate 10°C/min.

25°C to 100°C which we attribute to the loss of molecular water. There is then a further fractional weight loss of 0.16 on heating to 800°C due to dehydroxylation. Above 800°C the weight is stable. The TGA data are consistent with the empirical formula of Co-S-H given in the main text (Equation 2), assuming that the thermal decomposition product is the Co pyroxene $CoSiO_3$ (or the stoichiometrically equivalent Co olivine Co_2SiO_4 + SiO_2) known to form at high temperatures[25,26]. We write the two-stage decomposition as

$$\mathrm{Co_6Si_6O_7(OH)_{22} \cdot 3\,H_2O \rightarrow Co_6Si_6O_7(OH)_{22} + 3\,H_2O} \tag{S2a}$$

$$\mathrm{Co_6Si_6O_7(OH)_{22} \rightarrow 6\,CoSiO_3 + 11\,H_2O.} \tag{S2b}$$

The corresponding fractional weight changes are 0.05 and 0.15, in good agreement with experimental observation. This provides further support for the Co-S-H empirical formula $Co_6Si_6O_7(OH)_{22} \cdot 3\,H_2O$.

S6 Sorption isotherms of CS and Co-S-H

Water-vapour sorption isotherms of CS-1 and Co-S-H are shown in Figure S5. Data were obtained using an Aquadyne DVS instrument, using small blocks of intact CS-1 material before Co treatment and after complete conversion to Co-S-H.

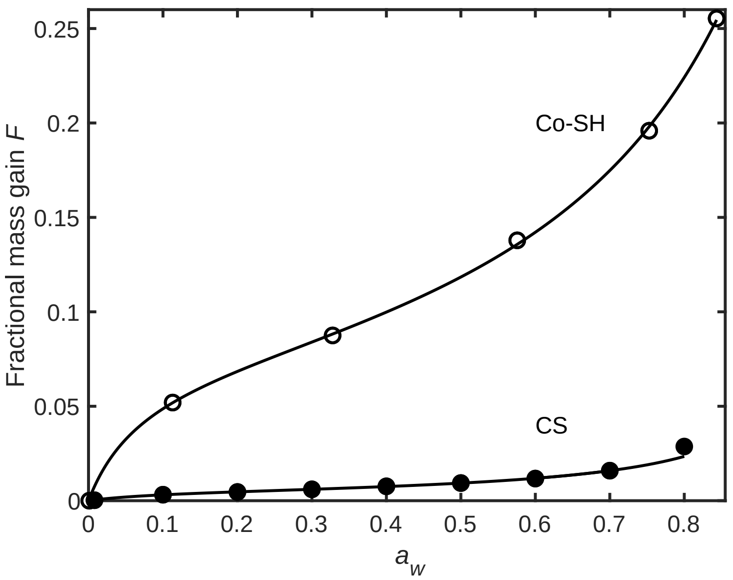

Figure S5 Water vapour sorption isotherms of CS and Co-S-H measured at 25 °C. The variation of fractional mass gain F with water vapour activity $a_\mathrm{w} = \mathrm{RH}/100$ is fitted (solid lines) to the Guggenheim-Anderson-De Boer (GAB) isotherm function $F = F_\mathrm{m} c_\mathrm{G} k_\mathrm{G} a_\mathrm{w} / \{(1 - k_\mathrm{G} a_\mathrm{w})[1 + (c_\mathrm{G} - 1)(1 + k_\mathrm{G} a_\mathrm{w})]\}$, where F_m is the fractional mass gain at monolayer coverage and c_G, k_G are the GAB parameters (CS: F_m $(5.222 \pm 0.353) \times 10^{-3}$, c_G 10.66 ± 2.68, k_G 0.979 ± 0.029; Co-S-H: F_m $(7.364 \pm 0.629) \times 10^{-2}$, c_G 16.46 ± 7.25, k_G 0.851 ± 0.029). Apparent GAB water-vapour surface areas are: CS, 19.4 ± 1.3 m^2 g^{-1}; Co-S-H 274 ± 23 m^2 g^{-1}.

S7 Filter-column sequestration experiments

The stirred-batch and diffusion-bed experiments described in the main text have been supplemented by filter-column experiments. There are similarities between the diffusion-bed and filter-column configurations, but in the filter column the Co solution percolates continuously at a controlled and steady flow velocity u through the packed CS powder bed (length L_b, volume fraction porosity f_b, packing density ρ_b). Mass transfer is by advection rather than by diffusion. The Co mass flux in the bed $j_\mathrm{m} = c'_\mathrm{s}u$, so that at the bed surface $j_\mathrm{m0} = c'_\mathrm{s0}u$. The zero-order Co sequestration kinetics allow us to write the differential change in c'_s along the bed as $\mathrm{d}c'_\mathrm{s} = -M_\mathrm{Co}k\rho_\mathrm{b}\mathrm{d}x = -k'\mathrm{d}x$. Hence, provided that the bed surface (at $x = 0$) has not reached sequestration capacity,

$$c'_\mathrm{s} = c'_\mathrm{s0} - \frac{k'}{u}x. \qquad \text{(S3)}$$

The mass concentration of Co^{2+} in solution c'_s falls linearly along the bed from the bed surface at $x = 0$ to a position $x_0 = uc'_\mathrm{s0}/k'$ where it is zero. If $x_0 < L_\mathrm{b}$ the filtrate at the exit at $x = L_\mathrm{b}$ contains zero Co, and this true for all flow velocities $u < L_\mathrm{b}k'/c'_\mathrm{s0}$. The region $0 \leq x \leq x_0$ defines a stationary reaction zone in which all incoming Co is sequestered. Knowing u and c'_s0 allows the sequestration rate constant k' (and hence k) to be calculated from the observed value of x_0.

At sufficiently long time, the bed surface reaches sequestration capacity m_cap (maximum kg Co/kg CS). This occurs at time $t = t_\mathrm{cap0} = m_\mathrm{cap}\rho_\mathrm{b}/k'$, where $t = 0$ is the time of first contact of the solution at the bed surface. At times $t > t_\mathrm{cap0}$, the reaction zone of constant length x_0 moves along the bed at velocity u. Co first appears in the filtrate (Co breakthrough) when the leading edge of the reaction zone reaches the end of the bed.

Table S6 Estimates of sequestration rate constant k from filter-column tests

		Test A	Test B
$\rho_{\rm b}$	$\mathrm{kg\,m^{-3}}$	185±15	210±7
$c'_{\rm s0}$	$\mathrm{kg\ Co\,m^{-3}}$	0.921±0.009	1.326±0.013
u	$10^{-6}\,\mathrm{m\,s^{-1}}$	1.80±0.45	1.15±0.30
x_0	$10^{-3}\,\mathrm{m\,s^{-1}}$	6.0±1.5	5.5±1.5
k	$\mathrm{mol\ Co\,(kg\ CS\ h)^{-1}}$	0.09±0.03	0.08±0.03

Note: Tests run at $22 \pm 1\,°\mathrm{C}$. The initial concentration of Co in solution is $c'_{\rm s0}$ (unit $\mathrm{kg\ Co\,m^{-3}}$ of solution).

Two filter-column experiments (Table S6) yield values of the rate constant k of 0.09 ± 0.02 and $0.08 \pm 0.03\,\mathrm{mol\ Co\,(kg\ CS\ h)^{-1}}$, in reasonable agreement with the value $k = 0.074 \pm 0.004\,\mathrm{mol\ Co\,(kg\ CS\ h)^{-1}}$ obtained in the stirred batch tests and which has smaller uncertainty.

S8 Gravimetric sequestration data

Table S7 shows raw weight data on CS blocks that have reacted completely

Table S7 Mass change of CS blocks on complete reaction with Co

Sample	Sample wt (g) before reaction	Sample wt (g) after reaction	Sample wt (g) after drying
A	1.1974	1.7755	1.6844
B	0.4853	0.7195	0.6871
C	0.9858	1.4171	1.3498
D	0.3966	0.5758	0.5470

Notes: (1) Samples were conditioned over LiCl saturated solution at 25.0 °C (RH 11.3 percent [27]) before and after reaction and prior to weighing. Dry weights were measured after conditioning the reacted samples over molecular sieve 4A at 25 °C (RH < 0.1 percent [28]).

with excess aqueous $Co(NO_3)_2$ solution at 25 °C. Uncertainty in weights is $\approx 0.0009\,\mathrm{g}$, arising from small random variations in adsorbed water content.

S9 Co sequestration rate: dependence on weight of CS used

In the analysis of the Co sequestration kinetics in the main text it was assumed that the rate of sequestration (mol/h) was proportional to the amount of CS used. This assumption, although reasonable, was not tested in the stirred batch experiments reported in the main text. In these experiments, a fixed amount of CS (1.00 g) was used in all runs.

In order to test the assumption, an additional series of stirred-batch tests was carried out in which the amount of CS used was varied from 0.10 g to 1.80 g while the quantity of solution remained constant at 25.00 g. The concentration of the solution was adjusted to ensure that in all runs there was sufficient CS to achieve complete removal of Co from solution. The amount of Co initially present in solution is denoted n_{Co}, as in Eqn 3 of the main text. In these tests, the clearance time for Co removal, t_c, was measured as the time at which the supernatant solution became colourless. Replicate runs, supported by thiocyanate colour tests for Co, show that t_c can be estimated with an uncertainty of at most 5 percent. In Fig S6 the quantity n_{Co}/t_c is plotted against the weight of CS used m_{CS}. The relation is linear over an 18-fold

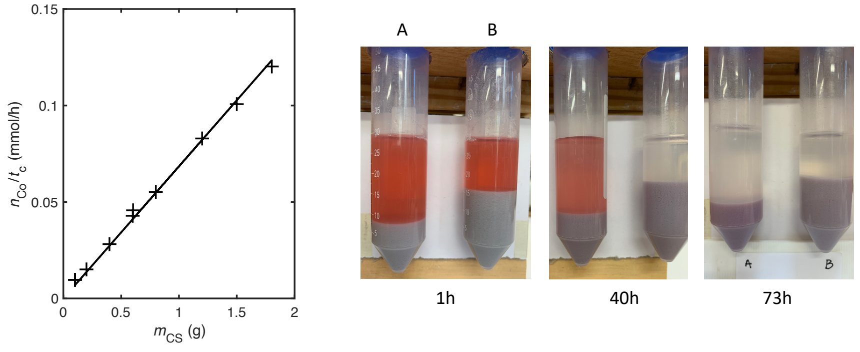

Figure S6 Rate of sequestation of Co by CS: variation with initial weight of Co m_{CS}. Photographs: tube A, 0.60 g CS, tube B 1.20 g CS; both tubes initially have 25.00 g 0.128 m $Co(NO_3)_2$ solution

variation of m_{CS} The slope of this line provides an estimate of the sequestration rate constant k. The value of 0.068 mol/(h kg CS) agrees well with the value of 0.074 mol/(h kg CS) given in the main text from earlier experiments, allowing for the fact that the CS used in these tests came from the Hamstad batch (CS-1) and has a larger particle size than CS-2.

S10 Solution densities

Densities of cobalt nitrate solutions calculated from[29] are given in Table S8.

Table S8 Aqueous Co(NO)$_3$ solutions at 25 °C: Interconversion of molality b, amount concentration c, and solution density ρ_{soln}.

Molality<br>b(Co)<br>mol kgw^{-1}<br>m	Amount concentration<br>c(Co)<br>mol L^{-1}	Density<br>ρ_{soln}<br>kg m^{-3}
0.050	0.050	1004.6
0.100	0.099	1011.9
0.150	0.149	1019.2
0.200	0.198	1026.4
0.250	0.247	1033.5
0.300	0.296	1040.6
0.400	0.393	1054.6
0.500	0.489	1068.4
0.600	0.585	1082.0
0.700	0.680	1095.5
0.800	0.774	1108.8
0.900	0.867	1122.0
1.000	0.959	1135.0

Note: Amount concentration $c = b\rho_{\mathrm{soln}}/(1 + bM)$ where the molar mass of Co(NO$_3$)$_2$ $M = 0.18294$ kg/mol.

References

[1] S. Roels, J. Carmeliet, H. Hens, O. Adan, H. Brocken, R. Cerny, Z. Pavlik, C. Hall, K. Kumaran, L. Pel and R. Plagge, *Journal of Thermal Envelope and*

Building Science, **2004**, *27*, 307–325.

[2] A. Hamilton and C. Hall, *Journal of Building Physics*, **2005**, *29*, 9–19.

[3] C. T. Do, D. P. Bentz and P. E. Stutzman, *Journal of Building Physics*, **2007**, *31*, 55–67.

[4] A. Hamilton and C. Hall, *Journal of Building Physics*, **2007**, *31*, 69–71.

[5] C. Hall, G. J. Lo and A. Hamilton, *Measurement Science and Technology*, **2022**, *34*, 027004.

[6] C. Hall and W. D. Hoff, *Water transport in brick, stone and concrete*, CRC Press, London and New York, 3rd edn, **2021**.

[7] Y. Kudoh and Y. Takéuchi, *Mineralogical Journal*, **1979**, *9*, 349–373.

[8] C. Hejny and T. Armbruster, *Zeitschrift für Kristallographie – Crystalline Materials*, **2001**, *216*, 396–408.

[9] K. Garbev, *PhD thesis*, Heidelberg University, **2003**.

[10] J. J. Esteban, J. Cuevas, J. M. Tubía and I. Yusta, *Canadian Mineralogist*, **2003**, *41*, 161–170.

[11] S. Bernstein, K. Thomas Fehr and R. Hochleitner, *Neues Jahrbuch für Mineralogie – Abhandlungen*, **2009**, *186*, 153.

[12] C. Dickson, D. Brew and F. Glasser, *Advances in Cement Research*, **2004**, *16*, 35–43.

[13] P. Blanc, X. Bourbon, A. Lassin and E. C. Gaucher, *Cement and Concrete Research*, **2010**, *40*, 851–866.

[14] P. Bots, M. Josick Comarmond, T. E. Payne, K. Gückel, R. J. Lunn, L. Rizzo, A. E. P. Schellenger and J. C. Renshaw, *Environmental Science: Processes and Impacts*, **2021**, *23*, 1101–1115.

[15] B. Ravel and J. J. Rehr, *Journal de Physique IV*, **1997**, *7*, 229–230.

[16] J. W. Gruner, *Zeitschrift für Kristallographie – Crystalline Materials*, **1934**, *88*, 412–419.

[17] F. Pertlik, *Monatshefte für Chemie*, **1999**, *130*, 1083–1088.

[18] J. P. Picard, G. Baud, J. P. Besse and R. Chevalier, *Journal of the Less Common Metals*, **1980**, *75*, 99–104.

[19] B. E. Warren and D. I. Modell, *Zeitschrift für Kristallographie – Crystalline Materials*, **1930**, *75*, 161–178.

[20] J. W. Gruner, *American Mineralogist*, **1934**, *19*, 557–575.

[21] K. Brauner and A. Preisinger, *Tschermaks mineralogische und petrographische Mitteilungen*, **1956**, *6*, 120–140.

[22] H. Evans and M. E. Mrose, *American Mineralogist*, **1977**, *62*, 491–502.

[23] A. Baldermann, V. Preissegger, S. Šimić, I. Letofsky-Papst, F. Mittermayr and M. Dietzel, *Cement and Concrete Research*, **2021**, *147*, 106521.

[24] B. Ravel and M. Newville, *Journal of Synchrotron Radiation*, **2005**, *12*, 537–541.

[25] S. R. Bohlen and A. Boettcher, *Geophysical Research Letters*, **1981**, *8*, 575–578.

[26] B. L. Brugman, M. Scharrer, T. S. Geraci and A. Navrotsky, *Materials Today Energy*, **2023**, 101382.

[27] L. Greenspan, *Journal of Research of the National Bureau of Standards. Section A, Physics and Chemistry*, **1977**, *81*, 89–96.

[28] A. Gorbach, M. Stegmaier and G. Eigenberger, *Adsorption*, **2004**, *10*, 29–46.

[29] B. S. Krumgalz, R. Pogorelsky and K. S. Pitzer, *Journal of Physical and Chemical Reference Data*, **1996**, *25*, 663–689.
